# Association of State Marijuana Legalization Policies for Medical and Recreational Use With Vaping-Associated Lung Disease

**DOI:** 10.1001/jamanetworkopen.2020.2187

**Published:** 2020-04-06

**Authors:** Coady Wing, Ashley C. Bradford, Aaron E. Carroll, Alex Hollingsworth

**Affiliations:** 1 O’Neill School of Public and Environmental Affairs, Indiana University, Bloomington; 2 Indiana University School of Medicine, Zionsville

## Abstract

This cross-sectional study evaluates the association between marijuana legalization policies and vaping-associated lung disease in states with recreational, medical, or prohibited marijuana use.

## Introduction

From June 2019 to January 2020, over 2500 cases of electronic cigarette (e-cigarette)– or vaping–associated lung injury (EVALI) were reported to the Centers for Disease Control and Prevention (CDC). The specific cause of EVALI is unknown, but most patients report using e-cigarettes to consume tetrahydrocannabinol (THC), the primary psychoactive component of marijuana. The CDC and others have hypothesized that black-market THC products may cause EVALI.^1,2^

Some states have legalized marijuana and THC-containing products for recreational use. Many other states allow purchases for qualifying medical purposes. In remaining states, all forms of consumption and distribution are illegal, and individuals who use THC likely obtain it from the black market. If black-market THC products are responsible for EVALI, then case rates may be lower in recreational marijuana states. The goal of this cross-sectional study was to measure whether states where marijuana is legal have lower rates of EVALI compared with states where it is illegal.

## Methods

We conducted a cross-sectional analysis at the state level plus Washington, DC (n = 51). We obtained data on the number of reported EVALI cases for each state in 2019 from the CDC,^2^ estimates of the prevalence of e-cigarette use in each state in 2017 from the Behavioral Risk Factor Surveillance System,^3^ and estimates of state populations in 2017 from the Surveillance, Epidemiology, and End Results database.^4^ This study was deemed exempt from approval by Indiana University's Institutional Review Board as it used publicly available, aggregate state-level data. We followed the Strengthening the Reporting of Observational Studies in Epidemiology (STROBE) reporting guideline.

We defined the EVALI case rate in each state as the midpoint of the CDC-reported range of cases per million population. We classified states as medical marijuana states if the state had a medical marijuana law by January 1, 2019, but no recreational dispensaries. We coded states as recreational marijuana states if the state had a recreational marijuana law and there was at least 1 recreational dispensary open in the state by January 1, 2019.

Throughout, the unit of analysis was the state, and all analyses are unweighted. We estimated a linear regression of the state EVALI case rate per 1 million people on indicator variables for recreational and medical marijuana states, leaving prohibition states as the reference group. The coefficients on the marijuana law variables are estimates of the difference in mean unadjusted EVALI case rates in recreational compared with prohibition states and medical compared with prohibition states.

Differences in e-cigarette use might confound the estimated association between EVALI and state marijuana laws if (1) the prevalence of e-cigarette use differed across states with recreational, medical, and prohibition laws and (2) the prevalence of e-cigarette use was correlated with EVALI rates. We investigated this possibility by fitting linear regressions of the state-level prevalence of e-cigarette use on indicator variables for recreational and medical marijuana laws. We also fit an augmented regression of EVALI case rates on the indicators for state marijuana laws and e-cigarette prevalence. All regressions used heteroskedasticity robust standard errors. We used 2-tailed *t* tests to assess the null hypotheses of no effect and rejected the null if the *P* value was less than .05.

## Results

**Figure 1.**
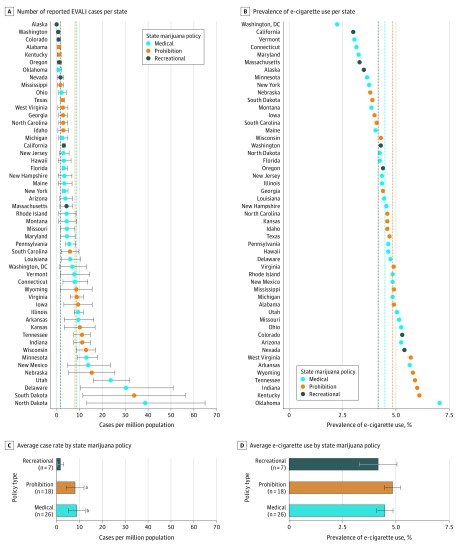
Rate of Electronic Cigarette (e-Cigarette)– or Vaping–Associated Lung Injury Cases and Prevalence of e-Cigarette Use by State and by State Marijuana Policy A state was considered to be a recreational marijuana state if it had at least 1 recreational dispensary open in January 2019. Results are robust to considering any state with a recreational marijuana law as of 2019 to be a recreational state. In A and B, brackets represent the range of EVALI cases per million, and dashed lines represent the mean by state marijuana policy. In C and D, brackets represent the 95% CI of each group mean. ^a^*P* = .003 compared with recreational. ^b^*P* < .001 compared with recreational.

Figure 1A shows the number of reported EVALI cases per million population in each state. Recreational marijuana states had among the lowest EVALI rates of all states. To test for differences in mean EVALI case rates across states with different marijuana policies, we regressed EVALI case rates on indicators for recreational and medical marijuana laws. The results are shown in Figure 1C. The average recreational marijuana state had 1.7 EVALI cases (95% CI, 0.3-3.1) per million population. In contrast, the EVALI case rate was 8.8 cases (95% CI, 5.1-12.5) per million population in medical marijuana states and 8.1 cases (95% CI, 4.1-12.0) per million population in prohibition states. A test of the difference in mean case rates implies that recreational marijuana states have 7.1 (95% CI, −10.9 to −3.2) fewer cases per million than medical marijuana states (*P* < .001) and 6.4 (95% CI, −10.4 to −2.3) fewer cases per million than prohibition states (*P* = .004). The difference in the EVALI case rate between medical and prohibition states was not statistically significant (difference = 0.7; 95% CI, −4.5 to 5.9; *P* = .78).

Figure 1B shows the prevalence of e-cigarette use in each state. To test for systematic differences in e-cigarette use, we regressed e-cigarette prevalence on marijuana law indicators. Figure 1D shows that the average e-cigarette use rate was quantitatively similar across the 3 groups of states, and none of the differences were statistically significant at conventional levels.

**Figure 2.**
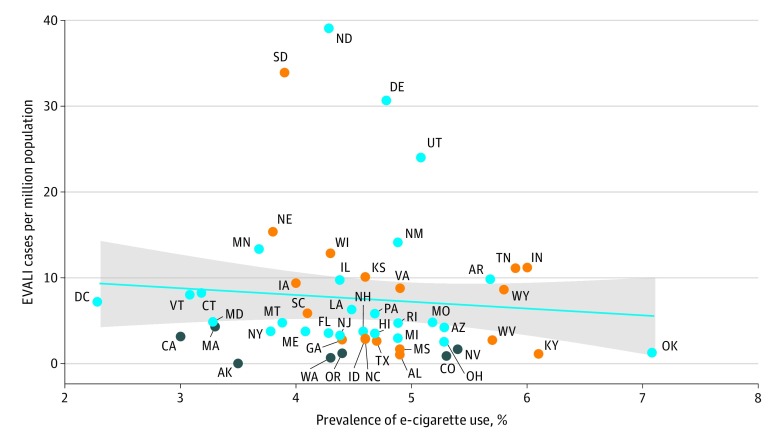
Electronic Cigarette (e-Cigarette)– or Vaping Case Rates vs e-Cigarette Use Rates Dark gray circles represent recreational marijuana states; orange circles, prohibition states; and blue circles, states that allow medical marijuana and do not have recreational dispensaries. A best-fit line is displayed in blue, with a slope of −0.8 and a robust standard error of 0.9 (*P* = .36). The gray shaded area denotes the 95% CI. Results are robust to weighting by state population.

Figure 2 shows a scatterplot of EVALI case rates against e-cigarette use rates. The graph suggests no association between EVALI cases rate and the prevalence of e-cigarette use in each state. We also used multivariable regression to estimate the association between the EVALI case rate and marijuana laws after adjusting for the prevalence of e-cigarette use. The results appear to confirm our earlier findings. The regressions imply that average EVALI case rates were lower in recreational marijuana states by 7.2 (95% CI, −11.8 to −2.6) cases per million population than in prohibition states (*P* = .003). There was no significant difference between EVALI case rates in prohibition and medical marijuana states (difference = 0.3; 95% CI, −5.3 to 5.8; *P* = .93). There was no association between the prevalence of e-cigarette use and EVALI case rates (difference = −1.3; 95% CI, −3.3 to 0.7; *P* = .20).

## Discussion

The data suggest that EVALI cases were concentrated in states where consumers do not have legal access to recreational marijuana dispensaries. This association was not driven by state-level differences in e-cigarette use, and EVALI case rates were not associated with state-level prevalence of e-cigarette use. One possible inference from our results is that the presence of legal markets for marijuana has helped mitigate or may be protective against EVALI.

The reason for this association is not yet clear. It is possible that in recreational states, people tend to purchase marijuana products at legal dispensaries, which may be less likely to sell the contaminated products that are thought to cause EVALI. In addition, the data are not informative about the potentially complicated interactions between safety regulations, bans, and prohibitions for goods such as marijuana, tobacco, and vaping products. Future research should examine these issues in more detail.

The statistical analysis and generalizability of results in this study have limitations. The data are aggregate state-level data and may not accurately reflect changes at the individual level. The results are based on simple cross-sectional comparisons and do not exploit an experimental or quasi-experimental research design that would mitigate concerns about the potential for confounding. The CDC data on EVALI cases by state provide the best available information about EVALI. However, they are reported as ranges rather than specific counts. There is also no way to know whether underreporting was a serious concern or whether underreporting varied across states.
